# A Novel Molecular Assembly of a Cobalt–Sulfate Coordination Polymer and Melamine: A Manifestation of Magnetic Anisotropy

**DOI:** 10.1021/acsomega.2c07556

**Published:** 2023-01-09

**Authors:** Ignacio Bernabé Vírseda, Shiraz Ahmed Siddiqui, Alexander Prado-Roller, Michael Eisterer, Hidetsugu Shiozawa

**Affiliations:** ‡ J. Heyrovsky Institute of Physical Chemistry, Czech Academy of Sciences, Dolejskova 3, 182 23Prague 8, Czech Republic; ¶ Faculty of Physics, University of Vienna, Boltzmanngasse 5, 1090Vienna, Austria; § Department of Inorganic Chemistry, University of Vienna, Währinger Straße 42, 1090Vienna, Austria; ∥ Atominstitut, TU Wien, Stadionallee 2, 1020Vienna, Austria

## Abstract

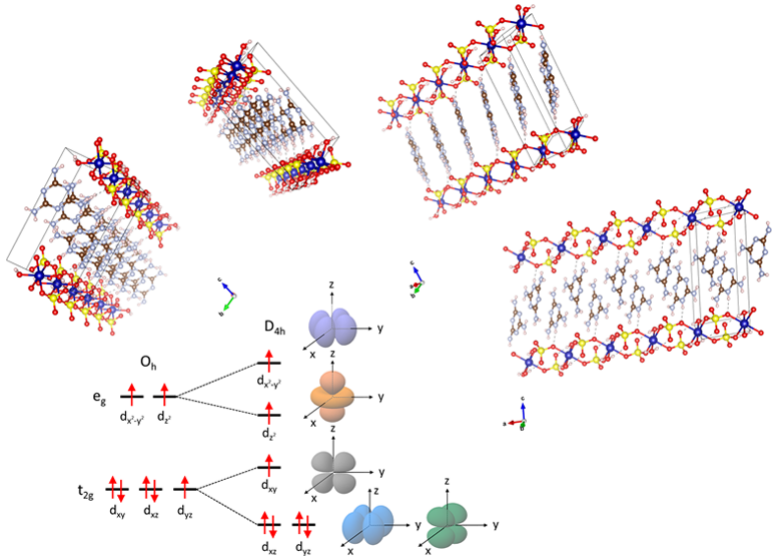

A novel molecular assembly of a cobalt–sulfate coordination polymer and melamine is synthesized under acidic conditions. Bar-shaped pink monocrystals as long as 1 mm are found to align along magnetic field lines in the proximity of a strong magnet. Magnetometry shows no hysteresis at temperatures down to 2 K but instead magnetic anisotropy and antiferromagnetic coupling. X-ray diffraction on a single crystal reveals that the cobalt–sulfate chains are along the shortest lattice vector or the crystal’s long axis. The crystal alignment along the magnetic flux can be attributed to single-ion anisotropy that results in longitudinal antiferromagnetic coupling along the chain. Both structurally and magnetically isotropic crystals of metal–organic hybrid materials can be highly useful as elemental components in magneto-optics.

## Introduction

Coordination polymers are molecular arrays composed of metal ions and bridging organic or inorganic ligands.^1−3^ Combinations of predesigned organic ligands and metal centers with versatile coordination geometries enable a variety of one-, two-, or three-dimensional lattice structures including porous metal–organic frameworks (MOFs). Because of their diverse properties, coordination polymers are promising materials for energy transfer, gas storage and separation, catalysis, electronic, optical, and magnetic applications.^4−6^

Molecular magnets are a class of magnetic materials based on organic molecules, coordination compounds, or hybrids. Coordination polymers as molecular magnets can exhibit various magnetic properties due to directional magnetic coupling among magnetic metals through low-dimensionally arranged molecular units. They are vital for understanding of the fundamentals of magnetic coupling and the magneto–structure correlation. In particular, those with higher critical temperatures could be useful in high-density magnetic information storage and quantum computation.^7−9^

**Figure 1:**
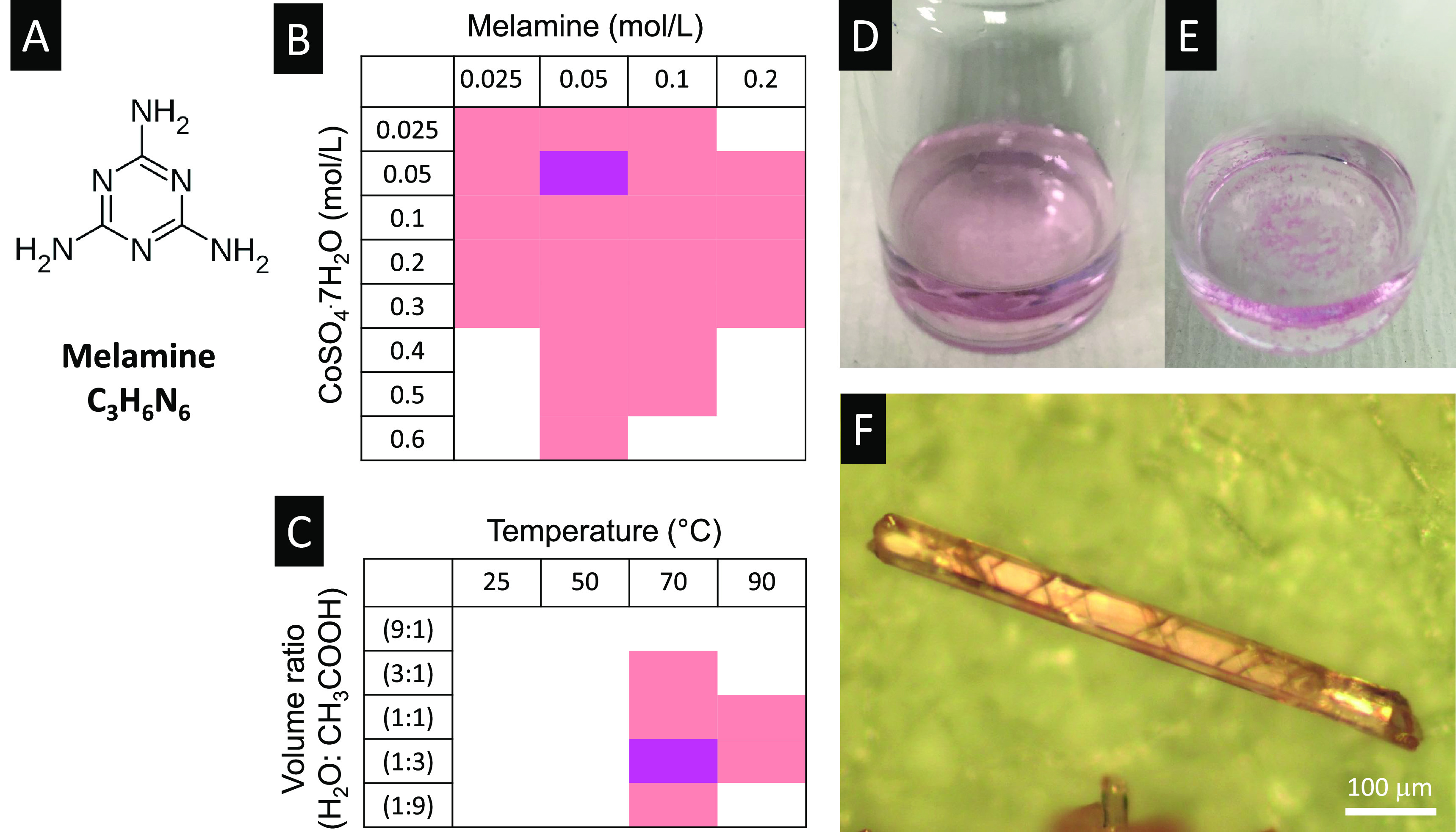
(A) The structure of melamine. (B) Crystal growth at 70 °C at various concentrations of CoSO_4_·7H_2_O and melamine. Purple and pink denote the best and marginal crystal quality, respectively, and white denotes no crystal formation. (C) Crystal growth at different temperatures and water to acetic acid ratios. (D and E) Photographs of the solution in the glass vial taken just as prepared and 3 h later, respectively. (F) Optical micrographs of CoS-M crystals synthesized under optimal conditions: 70 °C, (ρ_Co_, ρ_melamine_) = (0.05 mol/L, 0.05 mol/L), and a water-to-acetic acid volume ratio of 1:3.

In this contribution, a novel molecular framework of a cobalt–sulfate coordination polymer and melamine is synthesized. Melamine is a rich source of hydrogen bonds. Its molecular symmetry and skeleton rigidity (see Figure 1A) are useful for constructing supramolecular frameworks,^10^ while the use of melamine is not very common in coordination chemistry because of the low solubility in organic solvents.^11^ Synthesis parameters are optimized to yield millimeter-long single crystals. X-ray diffraction reveals layers of cobalt–sulfate chains packed with stacks of melamine by hydrogen bonding. Crystals align along the magnetic flux at room temperature like compass needles, making this polymer a good candidate for a molecular magnet. Magnetometry reveals no hysteresis but instead antiferromagnetic coupling at low temperatures and magnetic anisotropy. The weak but directional response of bar-shaped crystals to the external magnetic field can be attributed to the single-ion anisotropy that results in longitudinal antiferromagnetic coupling along the chain.

## Results and Discussion

### Synthesis

Unless otherwise noted, all crystals (referred to as CoS-M) have been synthesized by mixing an aqueous solution of CoSO_4_·7H_2_O and an acetic acid solution of melamine, followed by heating the mixture in a sealed glass vial.

The table in Figure 1B illustrates the quality of crystals synthesized at various concentrations of CoSO_4_·7H_2_O and melamine at 70 °C, which ensures the solubility of melamine in acetic acid. In each case, 1 mL of the aqueous solution of CoSO_4_·7H_2_O was placed in a 10 mL glass vial and then 3 mL of the acetic acid solution of melamine was added. The concentrations of CoSO_4_·7H_2_O in water tested are ρ_Co_ = 0.025 and 0.05, 0.1, 0.2, 0.4, 0.5, 0.6, and 0.8 mol/L, and the concentrations of melamine in acetic acid are ρ_Melamine_ = 0.025, 0.05, 0.1, and 0.2 mol/L. Purple and pink denote the best and marginal crystal quality, respectively, and white denotes no crystal formation. At ρ_Co_ = 0.8 mol/L, no crystals were formed. The largest crystals, approximately 500 μm long, as in Figure 1F, are obtained at (ρ_Co_, ρ_Melamine_) = (0.05 mol/L, 0.05 mol/L). When the sample is under the same conditions but pressurized in an autoclave (1.38 bar), the crystal length reaches around 1000 μm (see the Supporting Information (SI) for more details on the hydrothermal synthesis).

The higher concentrations hinder the formation of the crystals, possibly due to the increased ionic strength of both cations Co(II) and anions , which decreases the mobility and diffusion of the reactants and consequently affects the coordination between metal ions and the ligand.^12^ At the low concentrations, the mobility is high but nucleation does not occur.^13^

Figure 1C shows the quality of crystals formed at different temperatures and volume ratios of water and acetic acid. Temperatures tested are 25, 50, 70, and 90 °C, and volume ratios of water and acetic acid are (water/acetic acid) = (9:1), (3:1), (1:1), (1:3), and (1:9). The total volume of the solution was 4 mL.

The best-quality crystals grow at 70 °C and (1:3). At lower temperatures, melamine does not dissolve, as concluded in the solubility tests (see SI-1). As a result, no crystals are formed. This indicates that melamine plays a vital role in the formation of the crystal, ensuring its presence in the solid structure. At 90 °C, marginal quality crystals grow only at (1:1) and (1:3). A high temperature promotes the diffusion of reactants and consequently the nucleation. On the other hand, it hinders relaxation steps required for crystal formation.^14,15^ Therefore, temperatures higher than 70 °C are not optimal for the growth of CoS-M crystals.

As for the influence of the volume ratio, (water/acetic acid) = (1:3) is optimal. In the solvent with excess water (or acetic acid), melamine (or CoSO_4_·7H_2_O) does not fully dissolve.

Panels D and E in Figure 1 are the photographs of the solution in the glass vial taken just as prepared and 3 h later, respectively. The initial solution has pink color (Figure 1E) and a pH scale of 1.35 ± 0.06. The confidence interval was estimated as the 95% confidence interval of a “*t* of Student” distribution for which five simultaneous measurements were conducted. In approximately 30 min, pink dots appear on the bottom surface, and in 3 h the solution turns colorless as CoS-M crystals stop growing (Figure 1F). The pH scale at this point is 0.64 ± 0.08.

The fact that the solution gets more acidic after the formation of CoS-M may be related to the incorporation of H_2_O molecules into the crystals, which increases the concentration of acetic acid in the solution and consequently the concentration of H^+^.

### Structure Determination

**Figure 2:**
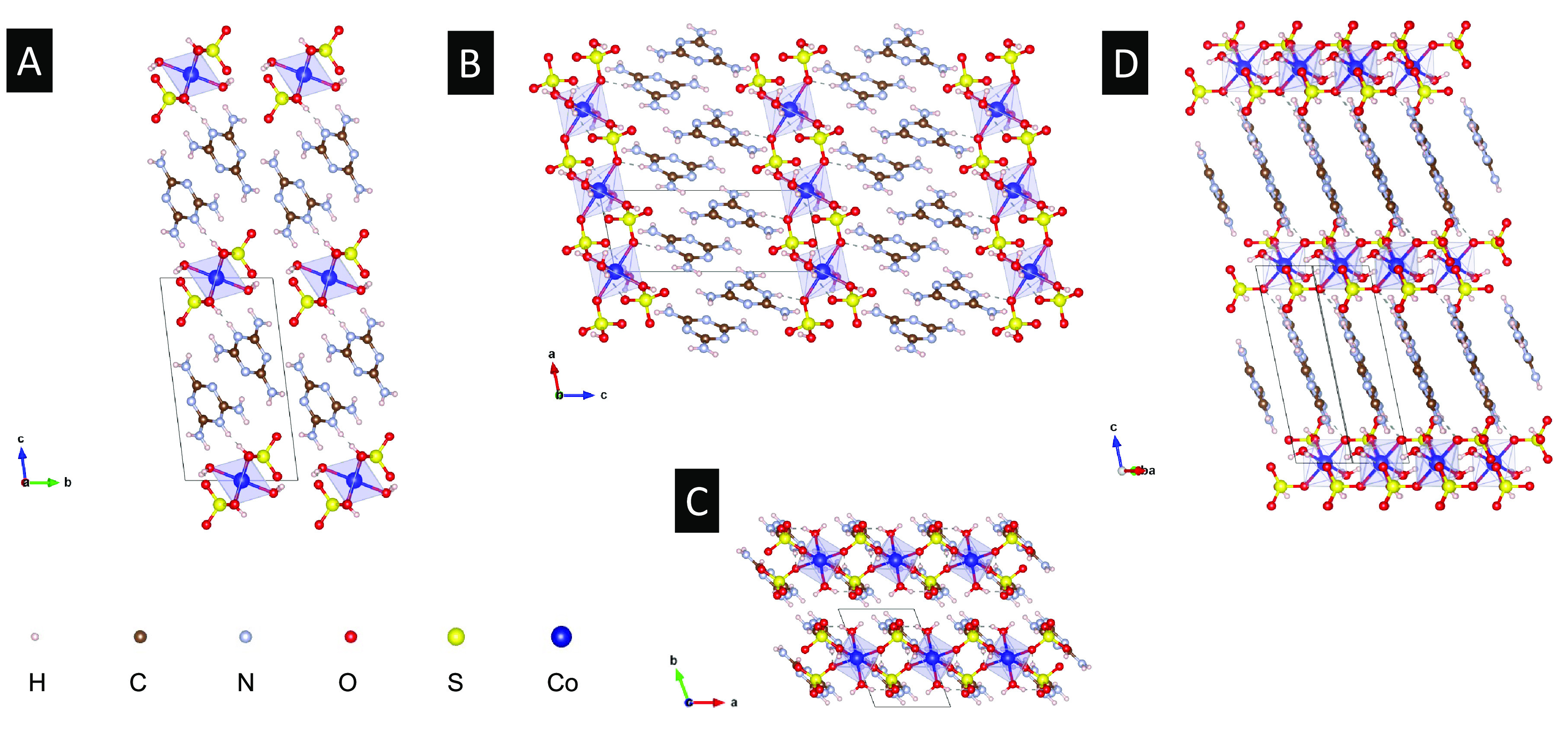
(a–D) The lattice structures of CoS-M viewed along axes *a*, *b*, *c*, and [1, −1, 0]. The space group is *P̅*1. The lattice parameters are *a* = 5.3182 Å, *b* = 7.2591 Å, *c* = 12.5757 Å, α = 93.3633°, β = 99.5922°, and γ = 109.6577°.

Single-crystal X-ray diffraction reveals that CoS-M is a coordination polymer that consists of cobalt cations, sulfate anions, water and melamine, as shown in Figure 2. The empirical formula of CoS-M is C_6_H_20_CoN_12_O_10_S_2_ . To the best of our knowledge, this structure has not been previously reported, and only a similar one was reported (CCDC 689784).^10^ The crystal is composed of cobalt–sulfate chains along the *a*-axis in which adjacent cobalt cations are bridged by two HSO_4_^–1^ anions through Co–O bonds. Two H_2_O molecules are also coordinated to the cobalt by their oxygen through Co–O bonds (2.08 Å). Each cobalt ion lies in an octahedral coordination geometry. The cobalt–sulfate chains lying in the (001) plane establish N–H···O bonds with melamine molecules, which are arranged in a zigzag fashion approximately within the (1 1 2) plane or facing the [2 2 1] direction. Importantly, the direction of the Co–S chains (axis *a*) was found to be along the long axis of the crystal.

### Magnetism

**Figure 3:**
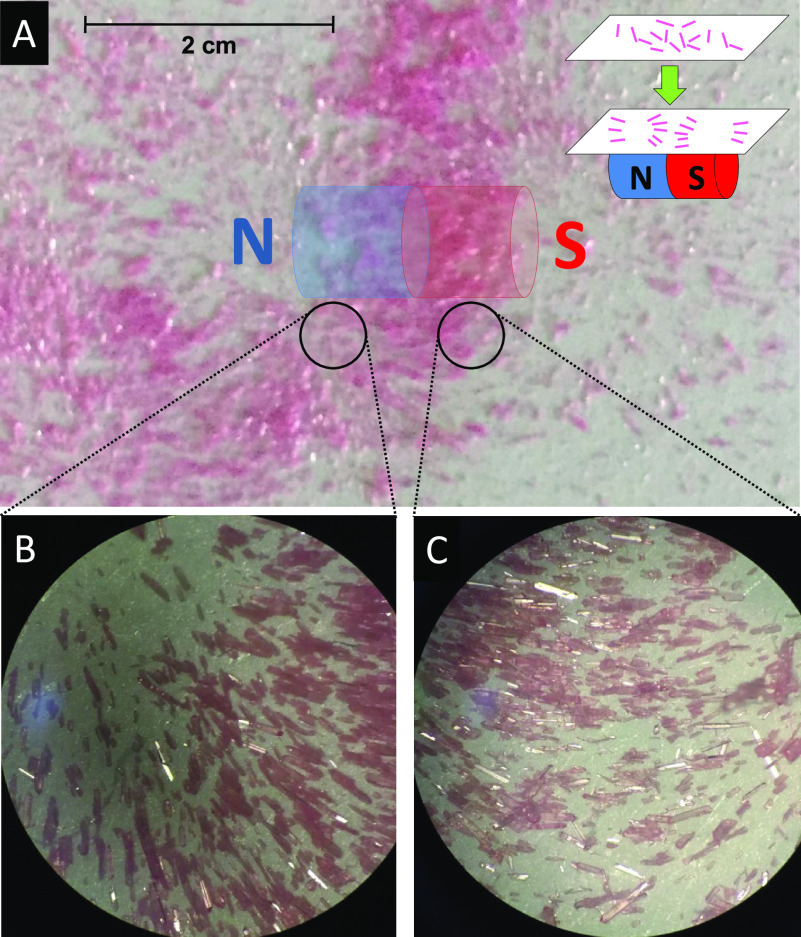
(A–C) Optical micrographs of the alignment of CoS-M crystals along magnetic fields. The inset in panel A shows a schematic diagram of CoS-M crystals dispersed on a paper and then subjected to the magnetic field of a cylindrical NdFeB magnet (VMM5H, Magsy, Czech Republic; diameter of 10 mm and length of 20 mm) underneath the paper.

The crystal’s orientations are susceptible to an external magnetic field. When exposed to strong magnetic fields of a neodymium magnet, the long axis of the crystal becomes aligned along the field direction, as shown in Figure 3. This means that the Co–S chains are aligned along the magnetic field, as the chain axis is along the crystal’s long axis. This longitudinal spin alignment is somewhat against the intuitive view that spins are aligned perpendicular to the chain axis and suggests the presence of magnetic anisotropy, which plays a decisive role in the orientation of crystals in a magnetic field.

The electronic configuration of divalent cobalt ion, Co(II), is [Ar] 3d^7^. The ligand field strength determines the splitting between the t_2g_ and e_g_ orbitals in an octahedral geometry. In a weak ligand field, the configuration is t_2g_^5^ e^2^, resulting in a high spin state with total spin *S* = 3/2. The ground-state term of this quartet state is ^4^T_1g_. In a strong ligand field, the configuration is t_2g_^6^ e^1^, i.e., a low-spin state with total spin *S* = 1/2. The ground-state term of this singlet state is ^2^E_g_.^16^

**Figure 4:**
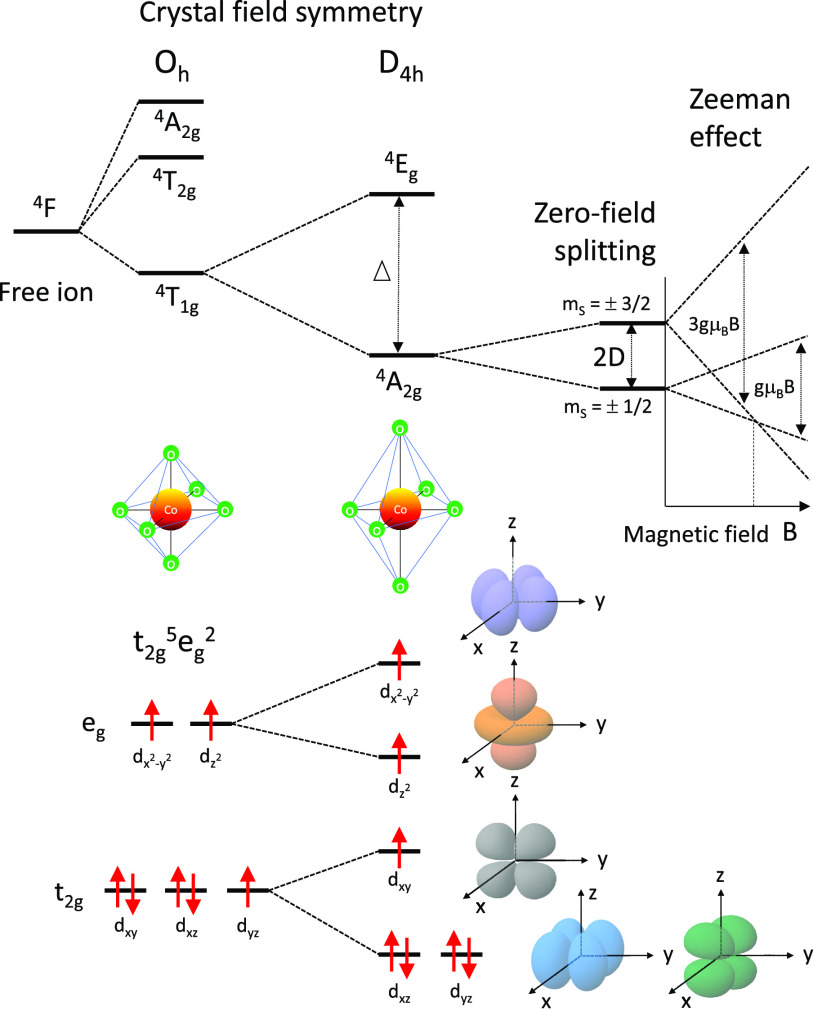
Energy diagram for a high-spin Co(II) ion in an axially distorted octahedral geometry.

A perfect octahedral coordination geometry O_*h*_ is hard to realize, and in most cases the octahedron is axially distorted to a tetragonal D_4*h*_ geometry. As shown in Figure 4, an axial elongation causes a splitting of the t_2g_ level, namely, the d_*xz*_ and d_*yz*_ orbitals move to a lower energy and the d_*xy*_ orbital moves to a higher energy. The e_g_ level splits into low-energy  and high-energy . The corresponding term symbols for the ground and first excited states are ^4^A_2g_ and ^4^E_g_, respectively, and are split by energy Δ. The ^4^A_2g_ state further splits into two Kramers doublets, *m*_*s*_ = ±1/2 and ±3/2, as a result of spin–orbit coupling, with the zero-field splitting (ZFS) energy denoted by *D* in Figure 4. The ground state is *m*_*s*_ = ±1/2 for *D* > 0 and *m*_s_ = ±3/2 for *D* < 0.

**Figure 5:**
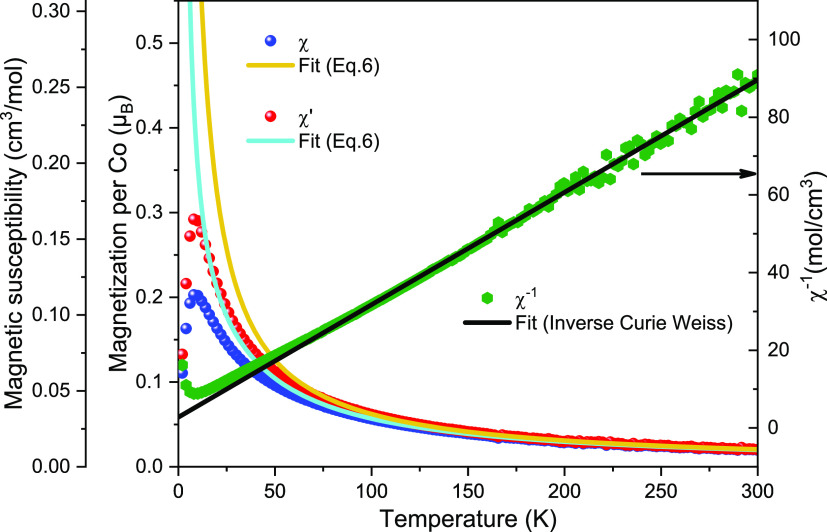
Magnetic susceptibility χ (solid blue circles),  (solid red circles), and the inverse magnetic susceptibility 1/χ (solid green hexagons) of CoS-M crystals measured in a magnetic field of 1 T in the temperature range of 2–300 K. The curves of best fit obtained for χ and χ′ in the temperature range of 100–300 K are extrapolated and plotted in cyan and orange, respectively, over the whole temperature range. The line of best fit obtained for 1/χ in the temperature range of 150–300 K is extrapolated and plotted over the whole temperature range.

Figure 5 shows the magnetic susceptibility χ (solid blue circles) and the inverse magnetic susceptibility 1/χ (solid green hexagons) of CoS-M crystals measured in a magnetic field of 1 T in the temperature range of 2–300 K. No hysteresis was observed between the zero-field cooled and field cooled data. See the SI for the χ*T* plot and the AC susceptibility.

At high temperatures, the susceptibility follows the Curie–Weiss law χ = *C*/(*T* – θ) where *C* is the Curie constant and θ the Weiss temperature. Using the inverse Curie–Weiss relation, the curve of best fit (the cyan curve in Figure 5) was obtained in the temperature range of 150–300 K with *C* = 3.45 cm^3^ mol^–1^ K) and θ = −9.53 K. The Curie constant is in agreement with the values for high-spin Co(II) in an octahedral environment (*C* = 2.8–3.4 cm^3^ mol^–1^ K) reported in literature.^17−27^ The corresponding effective magnetic moment is μ_eff_ = (8*C*)^1/2^ = 5.25. The negative Weiss temperature suggests an antiferromagnetic coupling, likely between the nearest neighbor spins of the CoS chains (see Figure 7). This is consistent with the deviation of χ downward from the Curie–Weiss law at temperatures below 150 K, leading to the peak at 8 K.

In order to further evaluate the spin ground state by taking the zero-field splitting (ZFS) (*D* in Figure 4) into account, we use the following spin Hamiltonian:^16^
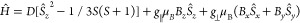
1where *D* is the ZFS parameter; , , and  represent spin operators; *S* is the total spin quantum number; *B*_*x*_, *B*_*y*_, and *B*_*z*_ are the three scalar components for the external magnetic field; *g*_∥_ and *g*_⊥_ are the *g*-tensors in the directions parallel and perpendicular to the *z*-axis, respectively,; and μ_B_ is the Bohr magneton. The second term in the expression is the spin Zeeman term.

The magnetic susceptibility in the parallel direction (χ_∥_) and that in the perpendicular direction (χ_⊥_) are derived as^28−41^
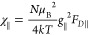
2
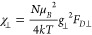
3where
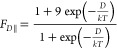
4
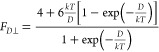
5*N* is the Avogadro constant, *k* is the the Boltzmann constant, and *T* is temperature.

The averaged magnetic susceptibility is given as
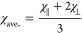
6We evaluate both χ and , namely, the susceptibility data with and without the Weiss molecular field term, which are plotted respectively as solid blue and red circles in Figure 5. The best fit for χ (orange curve in Figure 5) is obtained in the temperature range of 100–300 K, which gives *D* = 5.2 ± 0.49 meV (= 42.1 ± 3.95 cm^–1^), and g_ave._ = g_∥_ = g_⊥_ = 2.399 ± 0.006. The best fit for χ′ (cyan curve in Figure 5) obtained in the same temperature range leads to *D* = 0.97 ± 0.76 meV (= 7.8 ± 6.12 cm^–1^) and g_ave._ = g_∥_ = g_⊥_ = 2.405 ± 0.007. The zero-field splitting energy evaluated from χ′ is approximately five-times smaller than that evaluated from χ. As the Hamiltonian in eq 5 takes no exchange coupling into account, fitting χ′ should provide a better estimate of the upper limit of D.

The zero-field splitting energies *D* reported for cobalt complexes are in the range from −38 to +73 cm^–1^ when the coordination numbers are 5 and 4 and from +25 to +83 cm^–1^ when the coordination number is 6.^42,43^*D* = 0.97 ± 0.76 meV (= 7.8 ± 6.12 cm^–1^) obtained here from the χ′ is very small, approximately the thermal energy at 1 K. This means that in the temperature range of 100–300 K the zero-field splitting contributes to only a minor or no deviation from the S = 3/2 ground state.

**Figure 6:**
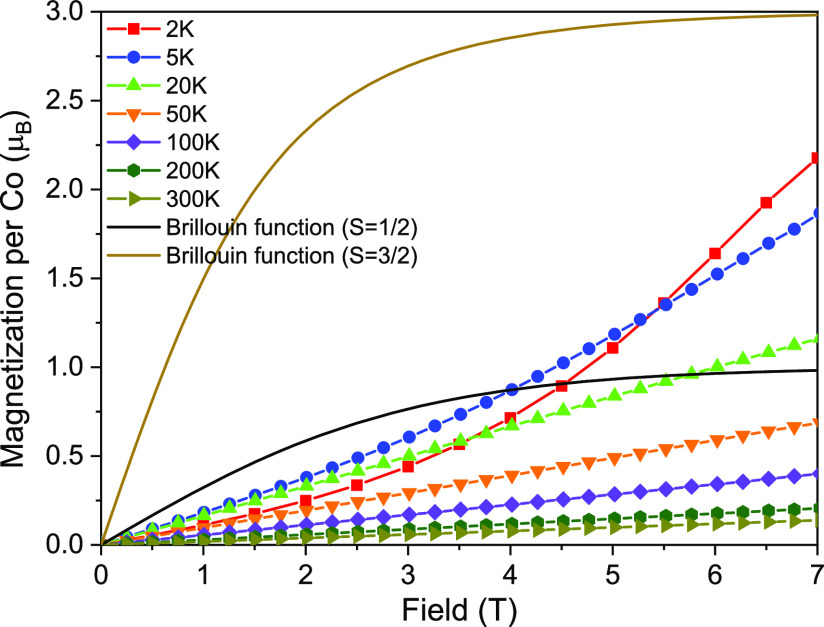
Magnetization isotherms measured at 2, 5, 20, 50, 100, 200, and 300 K.

Figure 6 shows the magnetization isotherms measured in fields up to 7 T at 2, 5, 20, 50, 100, 200, and 300 K. The low-temperature magnetism exceeds the saturation level (1.0 μ_*B*_/Co) of the Brillouin function for *S* = 1/2, supporting the *S* = 3/2 ground state at 2 K. The magnetization curve deviates significantly from the Brillouin function for paramagnetism and exhibits a sigmoidal shape. See the SI for the magnetization isotherms of roughly aligned crystals measured in fields up to 9 T both parallel and normal to the dominant crystal orientation.

The magnetic moment reaches a value of 2.17 μ_B_ at 7 T and seems to coincide with the saturation level (3.0 μ_B_/Co) for S = 3/2 in higher fields. The Zeeman energy in weak magnetic fields is proportional to ±(1/2)*g*μ_B_*B* for *m*_*S*_ = ±1/2 and ±(3/2)*gμ*_*B*_*B* for *m*_*S*_ = ±3/2.

The level crossing of the ground and first excited level separated by *D* = 0.97 ± 0.76 meV may occur in a magnetic field of ≥17 T, which is much higher than the measured magnetic field range. Additionally, this small zero-field splitting is visible only at temperatures below 1 K. Furthermore, the magnetization curve at 2 K is smaller than the Brillouin function for *S* = 1/2 in fields below 4.5 T. Hence, the sigmoidal magnetization curve can be attributed to the antiferromagnetic coupling of 3/2 spins in the Co–S chain.

**Figure 7:**
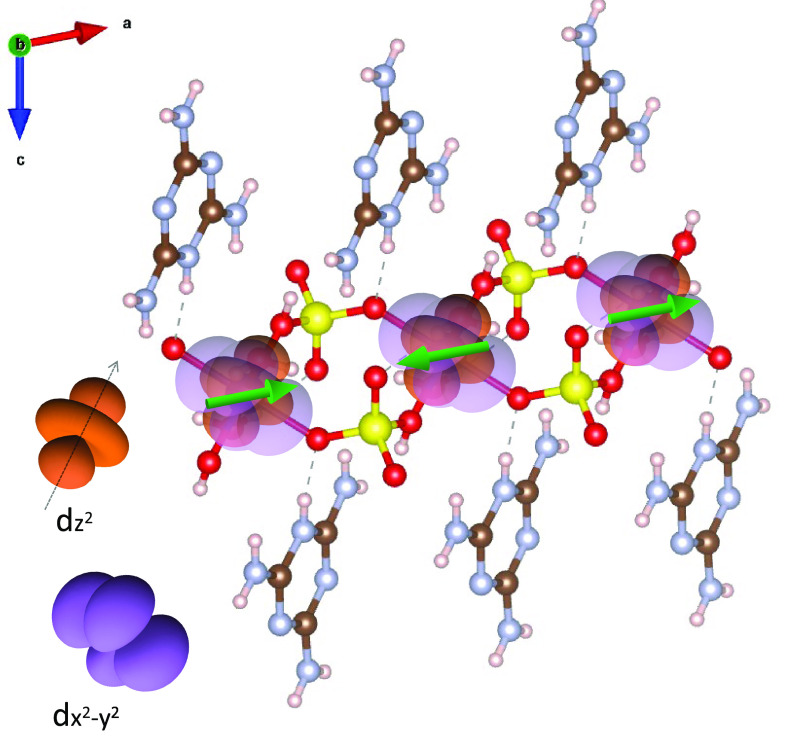
Co  and  orbitals with the quantization axis along the elongation direction of the octahedral ligand overlaid on the cobalt–sulfate coordination polymer lattice viewed along axis *b*. The green arrows represent cobalt spins antiferomagnetically coupled to one another and longitudinally aligned along the polymer chain axis.

As shown in Figure 3, the Co–S chains tend to become aligned along the magnetic field. This means that the spins are aligned along the chain and antiferromagnetically coupled one another, as depicted by the green arrows in Figure 7.

Now, we discuss mechanisms for the antiferromagnetic coupling. As shown in Figure 4, the three upper Co 3d orbitals, namely, 3d_*xy*_, , and  are half-filled and hence spin-polarized. The three spins are aligned one another according to Hund’s rule. The lobes of the Co  and  orbitals are along the Co–O ligands, while the 3d_*xy*_ orbital lies off the ligand. In Figure 7, half-filled Co  and  orbitals with the quantization axis along the elongation direction of the octahedral ligand are superposed onto the lattice. The distance between the nearest-neighbor cobalt atoms is 5.32 Å, which is too far for direct exchange. The superexchange coupling through the Co–O–S bonds with an angle of 133° may favor antiferromagnetic coupling between spins of the cobalt d orbital and the oxygen p orbital. However, the sp^3^ hybrid orbitals of sulfur in HSO_4_ have no favored exchange and should accommodate antiferromagnetically coupled spins in order to realize the antiferromagetic coupling of the adjacent cobalt ions. Hence, the antiferromagnetic coupling between cobalt atoms can be attributed to more elaborate interactions through super-exchange via HSO_4_, whose sign depends largely on the bonding environment but has been reported to be antiferromagnetic in many metal sulfates and related materials.

Finally, the origin of the longitudinal spin orientation could be the single-ion anisotropy, anisotropic diamagnetism due to the molecular orientation, or the demagnetizing field due to the crystal’s anisotropic shape. All melamine molecules face the [2 2 1] direction. Their aromatic rings exhibit anisotropic diamagnetism, which is the greatest when the rings face the field direction. The resulting demagnetizing field should favor the [2 2 1] direction along the field, but the crystals are aligned so as to make the [1 0 0] direction parallel to the field direction. In turn, the demagnetizing field for a typical crystal shape, e.g., 625 × 50 × 50 μm as in Figure 1F, does not exceed 20 mT, which is negligibly weak. Hence, the longitudinal orientation of the cobalt spins can be attributed to the single-ion anisotropy.

## Conclusion

A cobalt–sulfate coordination polymer and melamine are assembled to form millimeter-long magnetic monocrystals. X-ray diffraction on a single crystal reveals that the cobalt–sulfate chains are along the crystal’s long axis. Magnetometry evidences the longitudinal antiferromagnetic coupling of cobalt spins. As a result of the single-ion magnetic anisotropy, these magnetic crystals are aligned along magnetic field lines, which can be highly useful in magneto-optics.

## Supporting Information Available

CCDC 2173264 (CIF)
Experimental details, synthesis details, chemical and thermal stability, χ-plot, AC magnetic susceptibility, and anisotropy of the magnetic susceptibility (PDF)

## Supplementary Material

ao2c07556_si_001.cif

## Accession Codes

CCDC 2173264 contains the supplementary crystallographic data for this paper. These data can be obtained free of charge from The Cambridge Crystallographic Data Centre via www.ccdc.cam.ac.uk/structures.
