# Supplementary material for: A Novel Molecular Assembly of a Cobalt–Sulfate Coordination Polymer and Melamine: A Manifestation of Magnetic Anisotropy
Source: ACS Omega. 2023 Jan 9;8(3):3493–500. doi: 10.1021/acsomega.2c07556 (PMC9878548; doi:10.1021/acsomega.2c07556)
Supplement: Supplementary file 2 — ao2c07556_si_002.pdf [file ao2c07556_si_002.pdf]

Electronic Supplementary Information

A novel molecular assembly of a cobalt-sulfate coordination polymer and melamine: a manifestation of magnetic anisotropy

Ignacio Bernabé Vírseda,[a] **Shiraz Ahmed Siddiqui,**[b] **Alexander Prado-Roller,**[c] **Michael Eisterer,**[d] **and Hidetsugu Shiozawa**[a,b*]

[a]J. Heyrovsky Institute of Physical Chemistry, Czech Academy of Sciences, Dolejskova 3, 182 23 Prague 8, Czech Republic

[b]Faculty of Physics, University of Vienna, Boltzmanngasse 5, 1090 Vienna, Austria

[c]Department of Inorganic Chemistry, University of Vienna, Währinger Straße 42, 1090, Vienna, Austria

[d]Atominstitut, TU Wien, Stadionallee 2, 1020 Vienna, Austria

*To whom correspondence should be addressed; E-mail: hidetsugu.shiozawa@univie.ac.at & hide.shiozawa@jh-inst.cas.cz

1 Experimental

1.1 Chemicals

Cobalt (II) sulfate heptahydrate ($CoSO_4 \cdot 7H_2O$, 99%) and 1,3,5-Triazine-2,4,6-triamine (Melamine, $C_3H_6N_6$, 99%) were purchased from Sigma Aldrich. Acetic acid (CH_3COOH, 99.0 %) was provided from Penta and ultrapure water (18.2 MΩ·cm) was obtained from a water purifier (NANOpure Diamond, Werner, Germany). As for solvents used in the stability tests, hexane (C_6H_14, 99 %) dimethyl sulfoxide (DMSO, $(CH_3)_2SO$, ≥99.7 %), N,N Dimethylformamide anhydrous (DMF, $(CH_3)_2$-N-CHO, 99.8 %), 1-propanol (C_3H_7OH ≥99.7%), methanol (CH_3OH, ≥99.9 %) and ammonium hydroxide solution (NH_3OH, 28 % v/v in water) were purchased from Sigma Aldrich.

1.2 Optical microscope

Optical micrographs were taken using an optical microscope (BX41M-LED, Olympus, Japan) equipped with a Lumenera Infinity 1 camera and QuickPHOTO CAMERA 3.1 software.

1.3 Single crystal X-Ray diffraction

The X-ray intensity data were measured on Bruker D8 Venture diffractometer equipped with multilayer monochromator, Mo K/α INCOATEC micro focus sealed tube and Oxford cooling system.

1.4 Magnetization measurements

The magnetization data were recorded at temperatures between 2 K and 300 K in applied magnetic fields up to 7 T using a Quantum design MPMSXL magnetometer. 25.2 mg of crystals encapsulated in a gelatin capsule were measured.

2 Synthesis

Firstly, the solubility of melamine and cobalt (II) sulfate heptahydrate ($CoSO_4{\cdot}7H_2O$) was tested in hexane, water, methanol, DMF, DMSO, and acetic acid. The solutions with concentrations higher than 0.2 mol/L were prepared and homogenized in a Transsonic T 470/H ultrasonic bath. Melamine dissolved only in DMSO at room temperature and in acetic acid at 70 °C. On the contrary, $CoSO_4{\cdot}7H_2O$ showed good solubility in all the solvents except acetic acid.

Four solutions (0.2, 0.1, 0.05 and 0.025 mol/L) of melamine in acetic acid and eight solutions (0.8, 0.6, 0.4, 0.3, 0.2, 0.1, 0.05 and 0.025 mol/L) of $CoSO_4{\cdot}7H_2O$ in water, DMF, DMSO and methanol were prepared. Pairs of the above solutions were reacted at 25, 50, 70 and 90 °C in an oven (Ecocell model of BMT Medical Technology, Czech Republic). In all cases, the total volume was 4 mL. Among all the solvents tested, CoS-M crystals were formed only with aqueous solutions of $CoSO_4{\cdot}7H_2O$.

To evaluate the influence of the total volume of the solution, 0.05 mol/L aqueous solution of $CoSO_4{\cdot}7H_2O$ and 0.05 mol/L acetic acid solution of melamine were mixed with a volume ratio of 1:3. 2, 4 and 6 mL of the prepared solution were separately reacted at 70 °C. Panels A, B and C in Figure S1 are the optical micrographs of CoS-M crystals synthesized in the 2, 4 and 6 mL solutions. It is observed that the volume of the solution does not affect the crystal size which is approximately 400–500 μm in length.

2.1 Hydrothermal synthesis

Hydrothermal syntheses were performed using an autoclave with a 3 ml PTFE vessel. 0.05 mol/L aqueous solution of $CoSO_4{\cdot}7H_2O$ and 0.05 mol/L acetic acid solution of melamine were prepared. 0.5 mL of the aqueous solution of $CoSO_4{\cdot}7H_2O$ and 1.5 mL of the acetic acid solution of melamine were mixed, sealed in the vessel and heated at 70, 100, 140 and 180 °C in an oven.

The pressure in each experiment was estimated considering the vapor pressure of the solvents and the pressure of air. The latter can be estimated using Gay-Lussac's law:

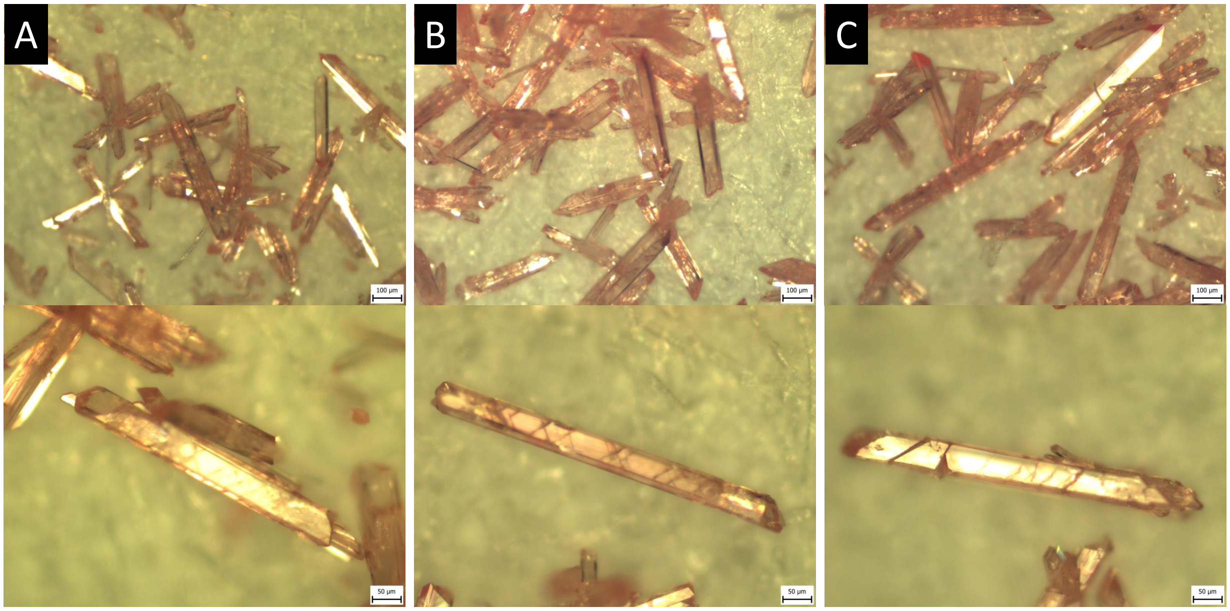

Figure S1: Optical micrographs of CoS-M crystals formed in 2mL (A), 4mL (B) and 6 mL (C) of the mixed solution (0.05 mol/L of $CoSO_4{\cdot}7H_2O$ in water, 0.05 mol/L of melamine in acetic acid, a volume ratio of 1:3).

$$\frac{P_1}{T_1} = \frac{P_2}{T_2}, \quad (1)$$

where P_i is the pressure at temperature T_i.

To estimate the vapor pressure of the mixture, the system is assumed to be an ideal mixture in which interparticle interactions are omitted Reid et al. [1959]. The vapor pressure of the mixture can be estimated using the Raoult's Law:

$$P_M = \sum P_{Vi} * X_i, \quad (2)$$

where P_M is the pressure of the mixture, P_{Vi} is the equilibrium vapor pressure of component "i", and X_i is the mole fraction of component "i" in the mixture.

The pressure of each individual component of the mixture was evaluated using the Antoine's law:

$$\log_{10} P_{Vi} = A - \frac{B}{T + C}, \quad (3)$$

where P_{Vi} (bar) is the equilibrium vapor pressure of component "i", T (°C) is the temperature of the media and A, B and C are component-specific constants.

Figure S2 displays the optical micrographs of the crystals obtained at different temperatures. It is observed that the crystal size becomes smaller as the temperature is increased.

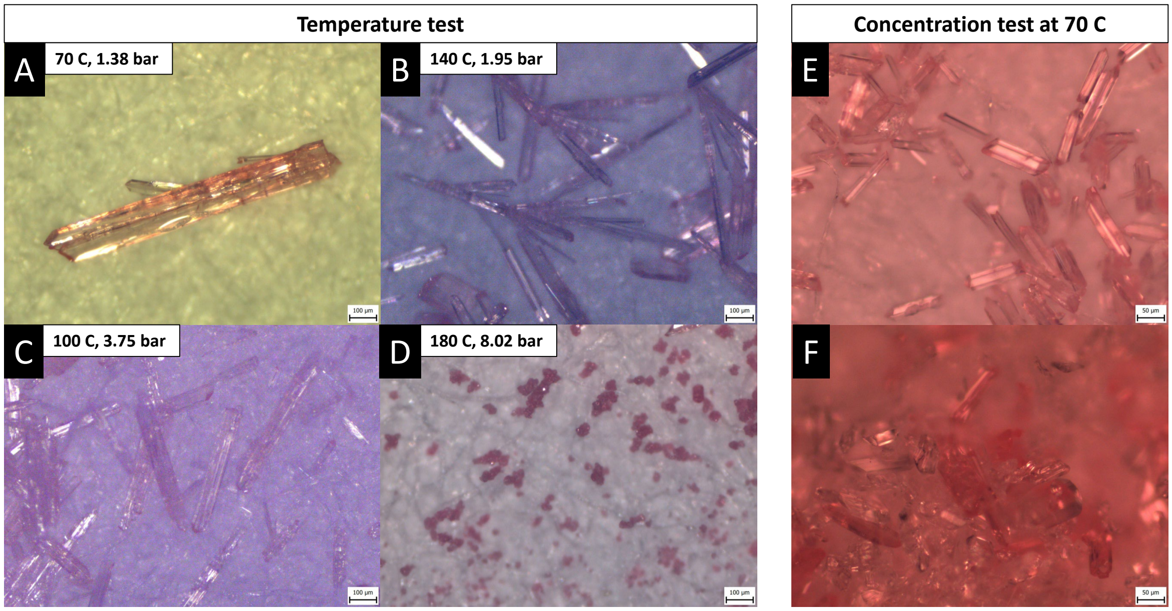

Figure S2: Optical micrographs of CoS-M crystals formed in the autoclave at temperatures of 70 °C (A), 100°C (B), 140 °C (C) and 180°C (D), labeled with the estimated pressures. Optical micrographs of CoS-M crystals formed in the autoclave at 70 °C with duplicated concentration (E) and triplicated concentration (F) of both $CoSO_4 \cdot 7H_2O$ and melamine.

Panels E and F in Figure S2 dislpay the optical micrographs of CoS-M crystals synthesized in the autoclave at 70 °C with concentrations of both reagents duplicated and triplicated, respectively, as compared to the case in panel A. Duplicating the reagent concentration reduces the crystal size from approximately 1000 to 200 μm in length. When the concentration is triplicated, the crystal length becomes as small as 50 μm. Also, transparent melamine crystals are precipitated.

3 Chemical stability

To test the chemical resistance to different solvents (hexane, DMSO, DMF, methanol, and purified water), crystals were filtered out and rinsed with 1-propanol, and subjected to 3 mL of a pure solvent in a closed glass vial of 5 mL volume. After a day, crystals were observed with an optical microscope and compared with those left in its forming solution.

To test the stability in acid and basic conditions, acetic acid and an aqueous solution of ammoniumhydroxide (NH_3OH, 28% v/v) were used. CoS-M crystals were washed and dryed before the test. For the acidic condition test, an acetic acid solution of pH 0.23 $\pm$0.11 was chosen because the pH of the mother solution after the crystal formation was 0.64 ± 0.08.

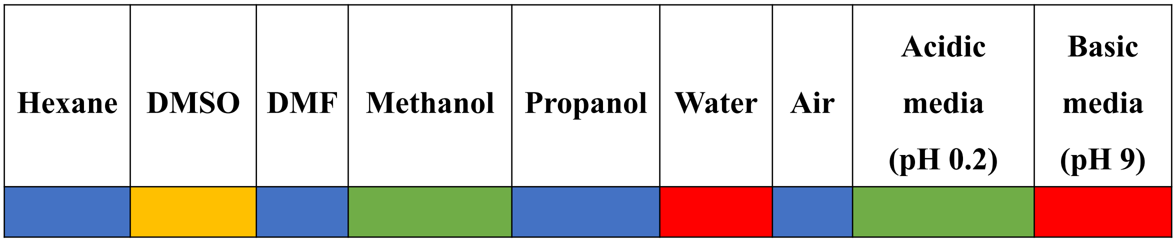

Red: decomposed in less than ten minutes; **Yellow**: decomposed in an hour;

Green: stable for a day; **Blue**: stable.

Figure S3: The stability of the CoS-M in air and various solvents

As summarized in Figure S3, the CoS-M is stable in air, propanol, DMF and hexane. In DMSO it decomposes in one hour and in water in less than 10 min. The basic condition boosts the decomposition, which might be caused by the replacement of linkers by OH^-. In the acid solution, the CoS-M stays stable for one day.

4 Thermal stability

For the thermal stability test, crystals were washed with 1-propanol and dried at room temperature. Each sample was subjected to a temperature-controlled atmosphere overnight in an oven (Ecocell model of BMT Medical Technology, Czech Republic). The temperature points tested were 100, 150, 200 and 300 °C. As shown in Figure S4, CoS-M crystals are stable at temperatures up to 150 °C above which the crystal colour changes from pink to violet.

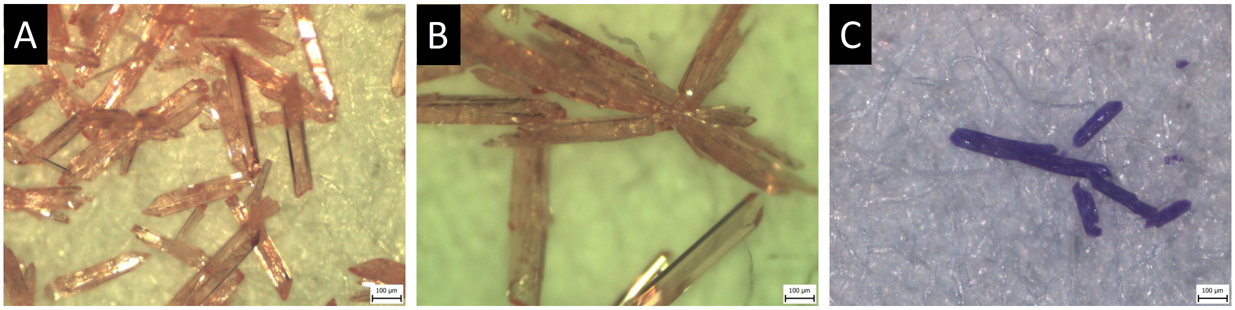

Figure S4: Optical micrographs of CoS-M crystals after being heated overnight at 100°C (A), 150 °C (B) and 200°C (C).

5 χT plot

Figure S5 shows the χT of CoS-M crystals measured in a magnetic field of 1 T in the temperature range of 2–300 K. The χT converges to 3.3 $cm^3 \cdot mol^{-1}K$ ($\mu_{eff} = (8\chi T)^{1/2} = 5.14$) at 300 K which is significantly higher than the spin-only value (1.875 $cm^3 \cdot mol^{-1}K$ for S=3/2) that can be attributed to the oribtal contribution. The orbital moment is often set as fictious or effective L = 1 for the $^4T_{1g}$ ground state using the T, P isomorphism.

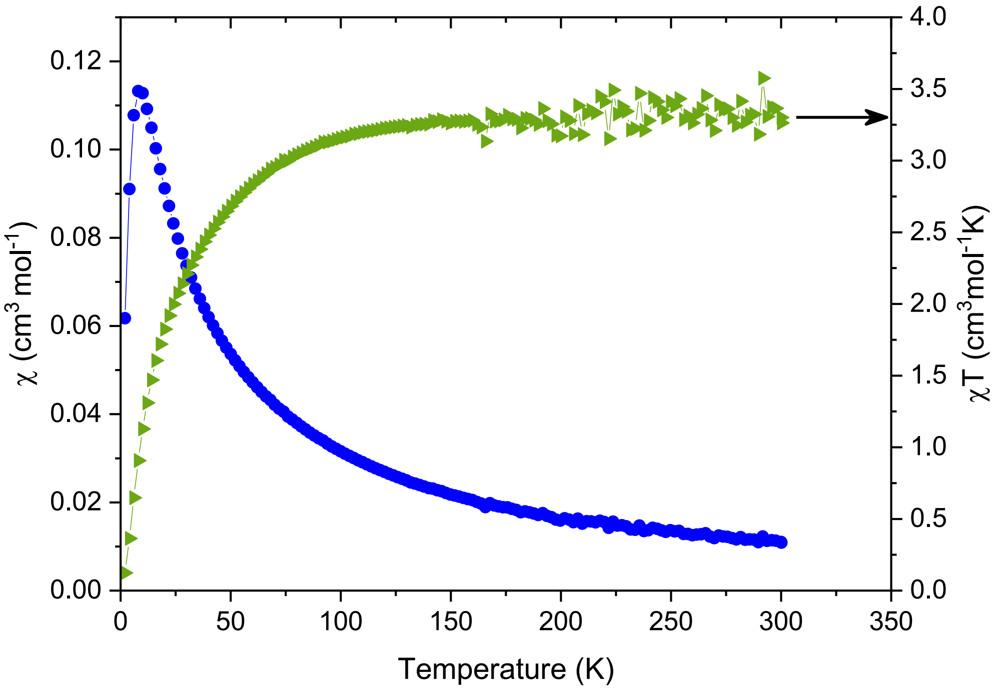

Figure S5: Temperature dependence of χ and χT for CoS-M crystals measured at 1T.

6 AC magnetic susceptibility

Panels A and B in Figure S6 show the real component (m') and imaginary component (m"), respectively, of the AC susceptibility of CoS-M crystals measured in zero DC magnetic field in the temperature range of 2–24 K with an AC amplitude of 3 Oe and frequencies in the range of 0.18 – 884 Hz. While the real component follows the DC susceptibility, the imaginary component is zero within the limits of experimental accuracy. It means that the relaxation time is slower than the measured frequency range.

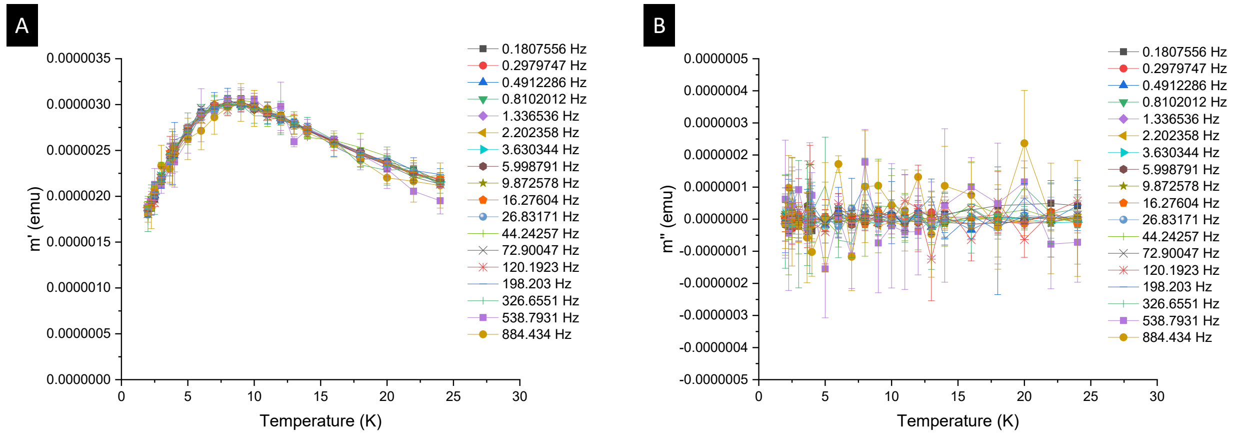

Figure S6: The real (A) and immaginary (B) components of the AC susceptibility for CoS-M crystals at different frequencies.

7 Anisotropy of the magnetic susceptibility

CoS-M crystals were aligned and encapsulated in polymethyl methacrylate (PMMA) by the following procedure. As-grown microcrystals were dispersed in an ethyl lactate solution of PMMA, drop-casted on a plastic tube and then placed on a flat Nd-Fe-B magnet surface. The sample on the magnet was placed in an oven at 140 °C to cure the PMMA.

The magnetic moments along and perpendicular to the crystal orientation were measured at 2 K in applied fields up to 9 T using a vibrating-sample magnetometer (Quantum Design Physical Property Measurement System). Based on the profile of the curve as shown in Figure S7 it is evident that the magnetization curve would reach a saturation value comparable to 3

μ_B/Co of the S = 3/2 state. The net magnetic moment of the CoS-M crystals oriented in the direction "parallel" to the field is greater by 8 % than that of the crystals "perpendicular" to the field.

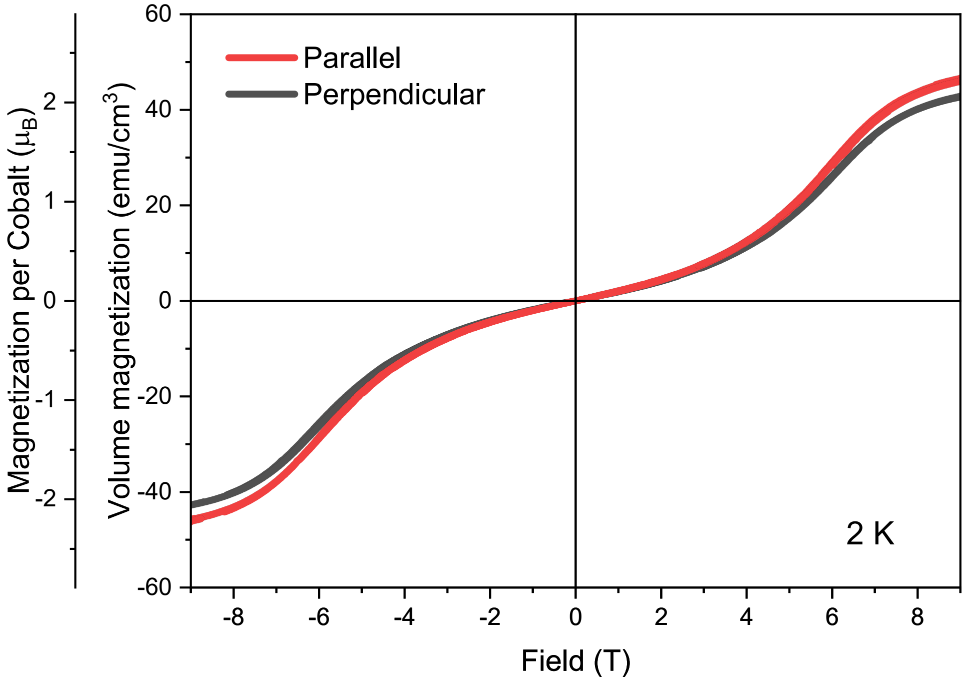

Figure S7: Magnetization isoltherms of CoS-M crystals aligned and encapsulated in PMMA measured at 2 K.

References

R. C. Reid, T. K. Sherwood, and R. E. Street. *The Properties of Gases and Liquids*, volume 12. 1959. ISBN 0071499997. doi:10.1063/1.3060771.
